# Iontophoretic Transdermal Delivery of Human Growth Hormone (hGH) and the Combination Effect of a New Type Microneedle, Tappy Tok Tok^®^

**DOI:** 10.3390/pharmaceutics10030153

**Published:** 2018-09-07

**Authors:** Gyubin Noh, Taekwang Keum, Jo-Eun Seo, Santosh Bashyal, Nyeon-Sik Eum, Min Jung Kweon, Sooyeun Lee, Dong Hwan Sohn, Sangkil Lee

**Affiliations:** 1 College of Pharmacy, Keimyung University, 1095 Dalgubeol-daero, Dalseo-gu, Daegu 42601, Korea; rhgyubin@naver.com (G.N.); gtk02@hanmail.net (T.K.); joeun0405@hanmail.net (J.-E.S.); bashyal.santosh18@gmail.com (S.B.); sylee21@kmu.ac.kr (S.L.); dhsohn@kmu.ac.kr (D.H.S.); 2 U-BioMed, 149-6 Yulam-Ro, Dong-Gu, Daegu 41059, Korea; ubiomed@daum.net (N.-S.E.); ubm333@daum.net (M.J.K.)

**Keywords:** recombinant human growth hormone, transdermal delivery, microneedle, iontophoresis

## Abstract

Transdermal drug administration presents several advantages and it is therefore favorable as an alternative drug delivery route. However, transdermal delivery of biopharmaceutical drugs is made difficult by the skin barrier. Microneedle application and iontophoresis are strategies which can be used to overcome this barrier. Therefore, recombinant human growth hormone (rhGH) was used as a model macromolecular drug and was transdermally delivered using microneedle application and iontophoresis. Methylene blue staining, stereomicroscopy and scanning electron microscope (SEM) imaging were used to characterize the microchannels produced. To optimize the iontophoresis protocol, the effects of molecular charge and current density on transdermal delivery were evaluated in an in vitro permeation study using excised rat skin tissues. Using the optimized iontophoresis protocol, the combination effects of iontophoretic delivery via microchannels were evaluated in three different experimental designs. The flux obtained with anodal iontophoresis in citrate buffer was approximately 10-fold higher that that with cathodal iontophoresis in phosphate buffered saline (PBS). Flux also increased with current density in anodal iontophoresis. The combination of iontophoresis and microneedle application produced higher flux than single application. These results suggest that anodal iontophoresis with higher current density enhances the permeation of macromolecules through microchannels created by microneedles. In conclusion, the combination of iontophoresis and microneedles is a potential strategy for the enhancement of transdermal delivery of macromolecular drugs.

## 1. Introduction

Administration of biopharmaceutical drugs such as proteins and peptides has been limited to invasive routes (e.g., intramuscular and subcutaneous injection), because of their poor absorption and enzymatic degradation. However, administration via invasive routes presents several disadvantages. First, the use of syringe needles causes pain and holds risk of infection such as needlestick injuries. Second, administration via invasive route is not suitable for patients with needle phobia. Third, invasive route administration induces financial and temporal costs, as patients generally need to visit a clinic for administration of medication. Even if self-administration is possible, patients should visit the clinic to learn the correct dosage regimen. As a result, these disadvantages can cause low patient compliance. Therefore, several studies investigating alternative routes of administration for macromolecules have been conducted to overcome these problems [1].

A potential alternative administration route that could solve this problem is the transdermal route. The skin has a relatively large surface area (1–2 m^2^), which is advantageous for drug administration. Transdermally administered drugs can avoid pre-systemic metabolism (e.g., degradation in the gastrointestinal tract and first-pass effect in the liver). It is also possible for patients to discontinue administration if desired, by removing the patch for example. However, the largest barrier for transdermal delivery of macromolecules is the stratum corneum, which is the outermost part of the skin. Because of the stratum corneum, only low-molecular weight drugs with moderate lipophilicity are transferred systemically via passive diffusion. For transdermal delivery of macromolecules, permeation enhancers are needed to overcome this barrier [2,3].

Iontophoresis, a second-generation transdermal delivery system, is a physical permeation-enhancing method, which uses an electrical driving force to enhance drug permeability of the biomembrane [2]. Iontophoretic delivery is based on the phenomenon of repulsion of same charges and attraction of opposite charges. The permeation-enhancing mechanisms of iontophoresis are electrorepulsion and electroosmosis [4]. The ability of low-molecular weight drugs to permeate through membranes is mainly governed by electrorepulsion. On the other hand, as the molecular weight of a drug increases, this ability is more affected by electroosmosis [5]. Iontophoresis is also known as a permeation enhancer capable of increasing peptide permeability in a non-invasive and controlled manner [6].

However, iontophoresis has limitations in that its permeation-enhancing effects are proportional to skin damage [2]. In addition, only molecules with a limited molecular weight can permeate via iontophoresis [7]. Therefore, the application of iontophoresis alone has its limitations, considering the difficult transdermal delivery of macromolecules.

A microneedle is a micron-sized needle that disrupts the stratum corneum and produces microchannels, allowing macromolecules and hydrophilic drugs to permeate through the skin [8]. Microneedle application is minimally invasive because of the needle’s size. It therefore affects only the stratum corneum, epidermis and superficial dermis when applied to the skin, does not affect nerve-endings in the dermis and enhances permeation of drugs without pain [2]. The permeation of drugs through microchannels is not limited by molecular weight, as the microchannels are micron-sized and the macromolecules are nanosized [7]. Microneedle application therefore allows not only small molecules but also macromolecules such as proteins and nanoparticles to permeate through the skin [9,10].

**Figure 1:**
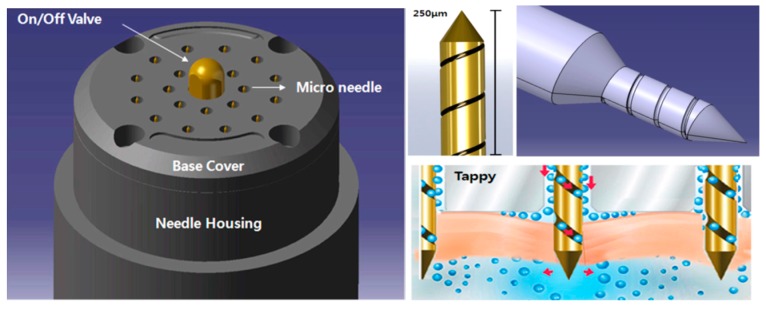
Schematic diagram of the microneedle device (Tappy Tok Tok^®^). (Reproduced from Eum et al. J Biomed Eng Res 2012; 33; 202–206, with permission of The Korean Society of Medical and Biological Engineering [11].).

The Tappy Tok Tok^®^ microneedle (Figure 1) is designed to enhance transdermal drug delivery using a novel approach. The diameter of the microneedles used (150 μm) is similar to the thickness of a hair to minimize pain. The microneedle was designed to enhance drug delivery by its unique surface structure. The screw-shaped groove on the surface of the microneedle allows drug injection through the groove whilst applying the microneedle to the skin [11].

Because one of the limitations of iontophoresis is that it does not change the skin barrier substantially, it is used for transdermal delivery of macromolecules together with third-generation transdermal delivery systems such as microneedles, ultrasound and chemical enhancers that can disrupt the skin barrier [2,9,12,13,14,15]. Considering that microneedle treatment is an effective way to overcome the limitations of iontophoresis, the combination of two enhancers is a potential strategy for transdermal delivery of macromolecules. The possibility of transdermal delivery of macromolecules such as proteins using a combination of iontophoretic delivery through microchannels has been studied previously [9,13,15].

In this study, recombinant human growth hormone (rhGH) was used as a large-molecular weight model drug. Since rhGH has a large molecular weight of approximately 22 kDa, it is difficult to deliver it without the use of permeation enhancers. To investigate the possibility of transdermal delivery of macromolecules, the permeation-enhancing effect of iontophoretic delivery through microchannels made with a novel microneedle device (Tappy Tok Tok^®^) on transdermal delivery of rhGH was evaluated.

## 2. Materials and Methods

### 2.1. Materials

Microneedle devices (Tappy Tok Tok ^®^) were developed and supplied by U-biomed, Inc. (Daegu, Korea). rhGH was obtained from Dong-A ST, Inc. (Yong-in, Korea). Silver wire was purchased from Sigma-Aldrich (St. Louis, MO, USA). rhGH enzyme-linked immunosorbent assay (ELISA) kits were purchased from R&D systems, Inc. (Minneapolis, MN, USA). All other chemicals and solvents were of reagent grade.

### 2.2. Microneedle Device (Tappy Tok Tok^®^)

The microneedle device consists of a head part comprising 20 microneedles on its surface and a bottle part that can hold drug solution. On the surface of the head, there is an On/Off valve and 20 microneedles arranged in a circle. The microneedles are made of stainless steel (SUS304) and gold (99.9%). Gold is coated on stainless steel microneedles to reduce metal allergic reactions in the skin. The On/Off valve controls drug elution when the microneedle device is applied to the skin after assembly of the head and bottom parts.

### 2.3. Skin Preparation

Rat skin tissues from male Sprague-Dawley rats (8 weeks old) were used for all permeation studies. The rats were anesthetized via intraperitoneal injection of urethane. The hair from the dorsal skin was removed using an electric clipper and then a depilatory was applied to remove any remaining hair. After detachment of the dorsal skin tissue, the underlying subcutaneous fat was carefully removed using forceps. The rat skin tissues were stored at −20 °C until further use. All experiments were performed according to guidelines approved by the Institutional Animal Care and Use committee of Keimyung University (KM 2018-002).

### 2.4. Characterization of Microneedles

To characterize microneedles and microchannels, stereomicroscopy and scanning electron microscope (SEM) imaging were performed. Rat skin tissues were placed on polystyrene plates and fixed with syringe needles, before microneedle application. The tissues were stained with 1% (*w*/*v*) methylene blue solution for visualization of the microchannels. After 1 min, excess methylene blue solution was removed using Kimwipes^TM^ and alcohol swabs and stereomicroscopy imaging was performed (SMZ-U, Nikon Corporation, Tokyo, Japan). The microchannels were observed via SEM (Hitachi S-4200, Hitachi, Ltd., Tokyo, Japan).

### 2.5. Skin Histology

A small area was cut from the rat skin tissue before microneedle application. The small area of tissue was fixed with optical cutting temperature (OCT) compound (Sakura Finetek, Tokyo, Japan) and frozen in liquid nitrogen. The block was cut into 8-μm-thick sections using cryostat (Cryotome FE, Thermo Fisher Scientific, Waltham, MA, USA) and mounted onto glass slides. Hematoxylin and eosin (H&E) staining was applied and cover slips were placed on the glass slides. Microscope imaging (Leica DM IL LED, Leica, Wetzlar, Germany) was then performed to visualize the creation of microchannels by application of the microneedle device.

### 2.6. Iontophoresis Protocol

For all iontophoresis experiments, the Ag/AgCl electrode was prepared using Jacobson’s method [16]. Briefly, Ag wire or a planar Ag electrode was soaked in distilled water, ethanol and fuming nitric acid 3 times for 3 s each. Next, the wire or planar electrode and another Ag wire (cathode) were dipped into 0.1 N HCl and a current of 1.0 mA was applied for 12 h to coat the AgCl.

Current was applied using a direct current (DC) power supply (UP-100DT, Unicorn, Gunpo, Korea) and measured using a digital multimeter (M-3610D, METEX, Seoul, Korea). The anode and cathode were placed in a donor chamber and sampling port according to the direction of iontophoresis.

### 2.7. In Vitro rhGH Permeation Study

**Figure 2:**
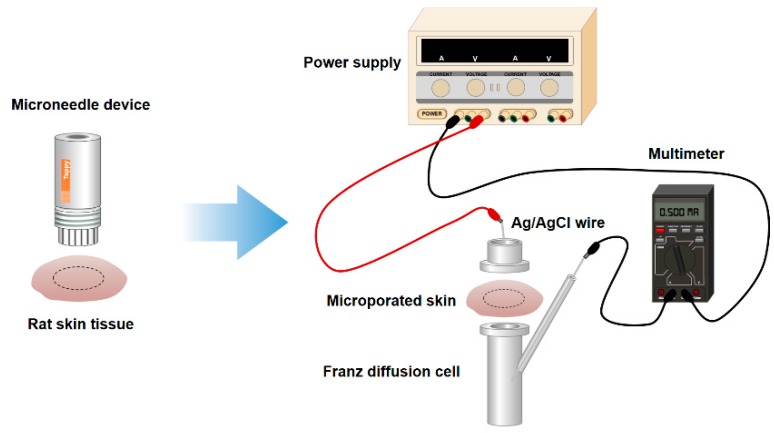
Schematic diagram of the in vitro recombinant human growth hormone (rhGH) permeation study.

As shown in Figure 2, an in vitro permeation study was performed on rat skin using a vertical static Franz diffusion cell with an effective area of 1.77 cm^2^. The receptor chamber was filled with PBS (pH 7.4) and the receptor medium was stirred constantly using a Teflon^TM^-coated magnetic stirrer at 600 rpm. For the microneedle treatment group, rat skins placed on a polystyrene plate and fixed with syringe needles were treated using the microneedle device for 1 min at a rate of two punctures per second with constant force. Next, treated skins were mounted on the receptor chamber with the epidermal surface facing the donor chamber. For the non-microneedle-treated group, intact skin was mounted. A 1-mL sample of rhGH solution was loaded into the donor chamber. During the permeation study, the Franz diffusion cell was maintained at 37 °C. Samples (0.5 mL) were taken from the donor chamber at pre-determined time points over 8 h and immediately replenished with an equal volume of PBS. Each experiment was performed in triplicate.

#### 2.7.1. Optimization of the Iontophoresis Protocol in rhGH Permeation

To evaluate the effect of molecular charge on rhGH, a permeation study investigating iontophoresis and microneedle treatment was conducted using two different buffers (PBS, pH 7.4 and citrate buffer, pH 4.0) to prepare a liquid rhGH formulation (isoelectric point = 5.27) with two different charges. Buffer exchange was performed using a centrifugal filter device (Amicon Ultra 0.5 mL, Millipore, Burlington, MA, USA) with a 3000-Da molecular weight cut-off. Because rhGH is negatively charged at pH 7.4 and positively charged at pH 4.0, formulations were delivered under cathodal and anodal iontophoresis, respectively. A constant current of 0.5 mA/cm^2^ was applied for 4 h through an Ag/AgCl electrode to both groups.

Furthermore, to evaluate the effect of current density on rhGH permeation, the permeation study on anodal iontophoresis and microneedle treatment was conducted using three different current densities. Current densities of 0.125, 0.25, or 0.5 mA/cm^2^ were applied for 4 h through Ag/AgCl electrodes.

#### 2.7.2. Combination Effects of Microneedle and Iontophoresis on rhGH Permeation

The combination permeation-enhancing effects of the optimized iontophoresis protocol with microneedle treatment were evaluated. Permeation studies were performed to evaluate the combination effects of iontophoretic delivery through microchannels under three different settings: (a) optimized iontophoresis alone (anodal iontophoresis with current density of 0.5 mA/cm^2^ for 4 h), (b) microneedle pre-treatment (1 min of application at a rate of two punctures per second with constant force) and (c) microneedle pre-treatment with optimized iontophoresis.

### 2.8. Analysis of rhGH

The amount of rhGH that had permeated was analyzed using a commercially available ELISA rhGH kit (DuoSet^®^, R&D system, Minneapolis, MN, USA). The standard curve ranged from 31.25 to 2000 pg/mL.

## 3. Results

### 3.1. Characterization of Microneedle

**Figure 3:**
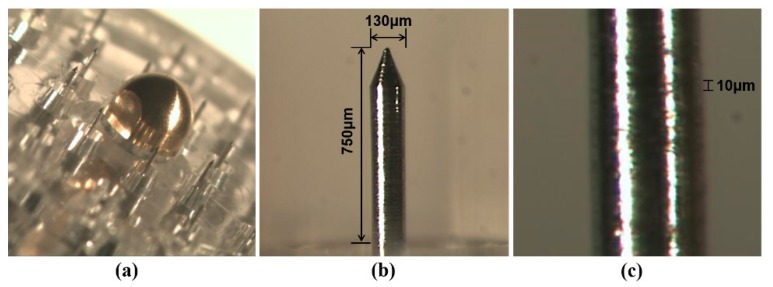
Picture of the head part of the microneedle device. (**a**) Head part, (**b**) microneedle and (**c**) surface of microneedle.

**Figure 4:**
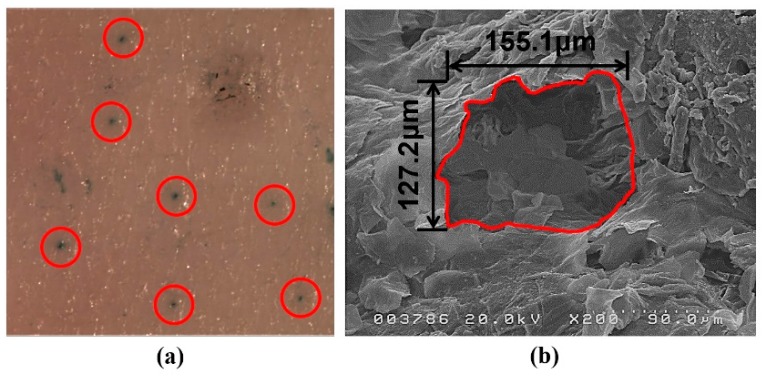
Images of rat skin tissue. (**a**) Stereomicroscope image after microneedle application, with methylene blue solution staining, (**b**) Scanning electron microscopy (SEM) image of rat skin tissue after application of microneedle device.

The microneedle device used in this study (Tappy Tok-Tok^®^) is made of stainless steel (SUS304). As shown in Figure 3, the device has 20 microneedles on its surface and each microneedle is 750 μm long and has a diameter of 130 μm. SEM imaging and methylene blue staining after microneedle treatment were conducted to characterize microchannels created by these microneedles. A picture of rat skin tissue stained with methylene blue after microneedle pre-treatment is shown in Figure 4a. Microneedles successfully generated microchannels in the rat skin tissue according to their arrangement. An SEM image of microchannels in rat skin tissue after microneedle treatment is shown in Figure 4b. A single microchannel has an area of approximately 0.016 mm^2^. Therefore, a single application of the microneedle device containing 20 needles disrupts a total of 0.32 mm^2^ of skin surface. In this permeation study, an area of 38.4 mm^2^ rat skin tissue was disrupted by applying Tappy Tok Tok^®^ for 1 min at a rate of two punctures per second. The effective diffusion area of the Franz diffusion cell used in the permeation study was 1.77 cm^2^. Therefore, Tappy Tok Tok^®^ theoretically disrupted approximately 21.7% of the effective diffusion area of rat skin tissue in this permeation study.

### 3.2. Skin Histology

**Figure 5:**
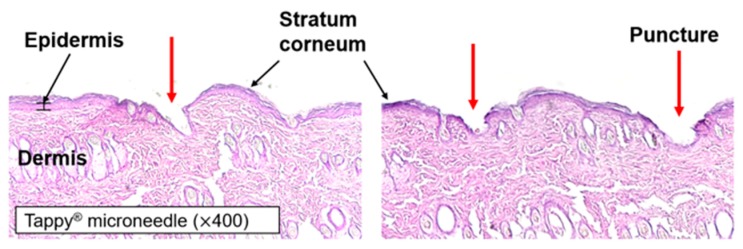
Histology of rat skin tissue after H&E staining.

As the application of microneedles causes structural changes in skin tissue, a histological study was conducted to confirm this effect. A histological representation of rat skin tissue after the application of the microneedle is shown in Figure 5. Microneedles penetrated the stratum corneum, epidermis and superficial dermis of rat skin tissue and produced microchannels. These microchannels allow macromolecules to be delivered through the skin by disrupting the stratum corneum. In addition, as the microneedle affects skin layers up to the superficial dermis only, it can enhance transdermal drug delivery without pain.

### 3.3. In Vitro rhGH Permeation Study

rhGH is a peptide hormone that is used in children and adults to treat growth hormone deficiency and growth disorders. rhGH is a single chain polypeptide comprising 191 amino acids and is difficult to deliver transdermally without the use of permeation enhancers because of its large molecular weight and hydrophilic nature [17].

The molecular limit of iontophoretic delivery through intact skin is approximately 13 kDa [7]. Because the molecular weight of rhGH is over this range (approximately 22 kDa), iontophoresis protocol optimization was conducted for use with microneedle pre-treatment. The steady-state rhGH flux was calculated from the slope of the linear portion of cumulative rhGH amount permeated over time [18].

#### 3.3.1. Optimization of the Iontophoresis Protocol in rhGH Permeation

**Figure 6:**
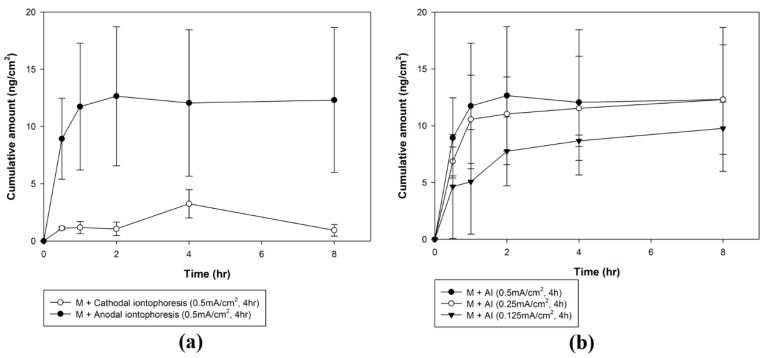
Cumulative amount of rhGH delivered through rat skin tissue after microneedle pre-treatment and iontophoresis with (**a**) two different molecular charges and (**b**) three different current densities.

**Figure 7:**
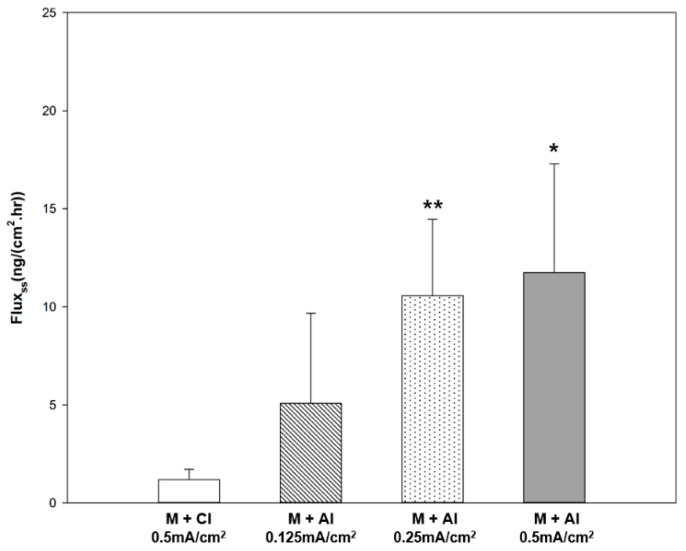
Steady-state flux of rhGH delivered through rat skin tissue after microneedle pre-treatment and iontophoresis with various protocols (* or ** indicates statistically significant difference (*t*-test; *p* < 0.05 or *p* < 0.01, respectively) compared with M + CI).

We investigated the iontophoretic delivery of rhGH through microchannels using two different molecular charges. rhGH is negatively charged above its isoelectric point and positively charged below it. Thus, negatively charged rhGH in PBS buffer (pH 7.4) was delivered under cathodal iontophoresis and positively charged rhGH in citrate buffer (pH 4.0) was delivered under anodal iontophoresis. The cumulative amount of rhGH permeated through the rat skin tissue by combination of cathodal or anodal iontophoresis with microneedle treatment is shown in Figure 6a. The cathodal iontophoresis and anodal iontophoresis groups showed a cumulative amount permeated of 2.91 ± 1.77 and 12.70 ± 6.12 ng/cm^2^, respectively. The cumulative amount permeated in the anodal iontophoresis group was approximately 4.4-fold higher than that in the cathodal iontophoresis group. The cumulative amount permeated reached a plateau within 1 h. As shown in Figure 7, the steady-state flux was 1.18 ± 0.53 and 11.75 ± 5.53 ng∙cm^−2^∙h^−1^ under cathodal and anodal iontophoresis, respectively, with the same current density. The steady-state flux was approximately 10 times higher in anodal iontophoresis.

Furthermore, three different current densities (0.125, 0.25 and 0.5 mA/cm^2^) were applied to identify the correlation between current density and cumulative rhGH amount permeated. As shown in Figure 6b, the cumulative amounts permeated were 10.76 ± 0.9, 13.94 ± 4.5 and 12.70 ± 6.12 ng/cm^2^ at a current density of 0.125, 0.25 and 0.5 mA/cm^2^, respectively. There was no significant difference in cumulative amount of rhGH permeated with different current densities. However, steady-state fluxes at the initial permeation time of 1 h were significantly different. Steady-state flux was 5.07 ± 4.61, 10.57 ± 3.89 and 11.75 ± 5.53 ng∙cm^−2^∙h^−1^ at a current density of 0.125, 0.25 and 0.5 mA/cm^2^, respectively.

#### 3.3.2. Combination Effect of Microneedle Application and Iontophoresis on rhGH Permeation

**Figure 8:**
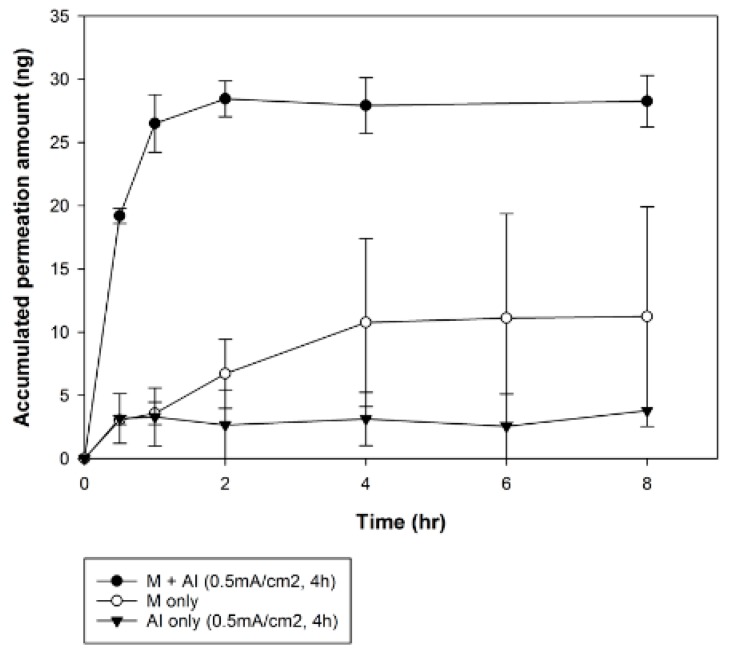
Cumulative amount of rhGH delivered through rat skin tissue after microneedle treatment alone, iontophoresis alone, or both in combination.

The combination effect of microneedle treatment and iontophoresis was studied under three different conditions. The cumulative permeated amounts were 1.89 ± 1.25, 6.63 ± 4.69 and 12.70 ± 6.12 ng/cm^2^, after iontophoresis only, microneedle pre-treatment only and microneedle pre-treatment with iontophoresis, respectively. The group receiving microneedle pre-treatment with optimized iontophoresis resulted in 1.92- and 6.73-fold higher cumulative amount permeated than the group receiving iontophoresis alone and microneedle pre-treatment alone, respectively. The combination of microneedle and iontophoresis resulted in a much greater permeation-enhancing effect than when each was applied separately. The synergetic effect of microneedle application and iontophoresis on permeation was also clearly observed in steady-state flux. The application of iontophoresis and microneedle, respectively, resulted in steady-state flux of 1.86 ± 1.30 and 2.02 ± 0.50 ng∙cm^−2^∙h^−1^, respectively and when used together, steady-state flux of 11.75 ± 0.50 ng∙cm^−2^∙h^−1^ was observed. The combination group resulted in 6.33- and 5.82-fold higher steady-state flux than iontophoresis alone and microneedle pre-treatment alone, respectively. The synergetic effect of increasing steady-state flux of rhGH with microneedle application and iontophoresis is shown in Figure 8.

## 4. Discussion

The transdermal route presents several advantages as an alternative route of administration: (1) skin is easily accessible because it is the outermost layer of the body and (2) it has a relatively large surface area (1–2 m^2^). However, because the skin basically acts as a barrier for molecular transport, only drugs which possess certain physico-chemical properties can penetrate without the help of penetration enhancers [19]. Microneedles allow for the transdermal delivery of macromolecules by painlessly creating microchannels in the stratum corneum, which is the main barrier of the skin [2]. Iontophoresis enhances delivery of peptides in a non-invasive and controlled manner [6]. In this study, we evaluated the possibility of transdermal administration of biopharmaceutical drugs using microneedle application and iontophoresis as penetration enhancers.

Through the characterization of microneedles, the micron-sized needle and the specific structure of the surface were observed. Methylene blue was not observed in the intact skin but it diffused into the skin through the microchannels produced by microneedle pre-treatment. SEM imaging revealed that the area of each microchannel was approximately 0.016 mm^2^. As shown in Figure 5, microneedles penetrated the stratum corneum and punctured the superficial dermis.

An in vitro rhGH permeation study using excised rat skin tissue was performed to optimize the iontophoresis protocol. The flux of iontophoretic delivery is theoretically calculated as the sum of passive delivery, electrorepulsion and electroosmosis [13]. The balance between electrorepulsion and electroosmosis is affected by the molecular size of the drug molecules. Iontophoretic transport of small molecules is mainly enhanced by electrorepulsion. In the case of electrorepulsion, relative flux decreases as molecular size increases, while electroosmosis maintains constant relative flux regardless of molecular size [20]. Therefore, the flux of electroosmosis becomes larger than that of electrorepulsion if the drug is larger than a certain size [5]. Because of its large size, electroosmosis is the main mechanism of iontophoretic delivery for rhGH. The electrorepulsive flow of negatively charged rhGH in cathodal iontophoresis competes with electroosmotic flow from anode to cathode [21]. The positively charged rhGH in anodal iontophoresis produced relatively high flux via the combination of electrorepulsive and electroosmotic flow. The skin is negatively charged, which is favorable for the permeation of cations [22]. As a result, positively charged rhGH with anodal iontophoresis resulted in cumulative amounts approximately 4-fold higher and steady-state flux 10-fold higher than that achieved with cathodal iontophoresis.

Application of constant current in iontophoresis results in time-dependent changes in the skin. Iontophoresis reduces the resistance of skin through reversible pore formation and enhances drug permeation. This change in skin occurs at a relatively low voltage (approximately 1 V) and within a short time (approximately 10 min after application of constant current). Furthermore, after the removal of current, the skin returns to its original state, although this is not completely reversible [23,24]. Application of constant current for a long time can induce electrochemical polarization of the skin, which increases its resistance and reduces the permeation-enhancing effect. Since iontophoresis was carried out for a relatively long time in this study using a high voltage, the cumulative permeation amount plateaued rapidly after 1 h.

As shown in Figure 7, the steady-state flux of rhGH increased with the current density. The approximate linear correlation between current density and steady-state flux is advantageous in that the amount of drug delivered can be controlled. The dose requirement of rhGH depends on the patient’s response to the hormone and the weight of the individual [25]. In addition, an iontophoretic protocol can be programmed with an electronic controller to allow the drug to be delivered according to normal physiological secretion patterns or to deliver drugs during sleep [6]. Therefore, personalized transdermal delivery of biopharmaceutical drugs will be made possible by controlling the iontophoresis protocol.

**Figure 9:**
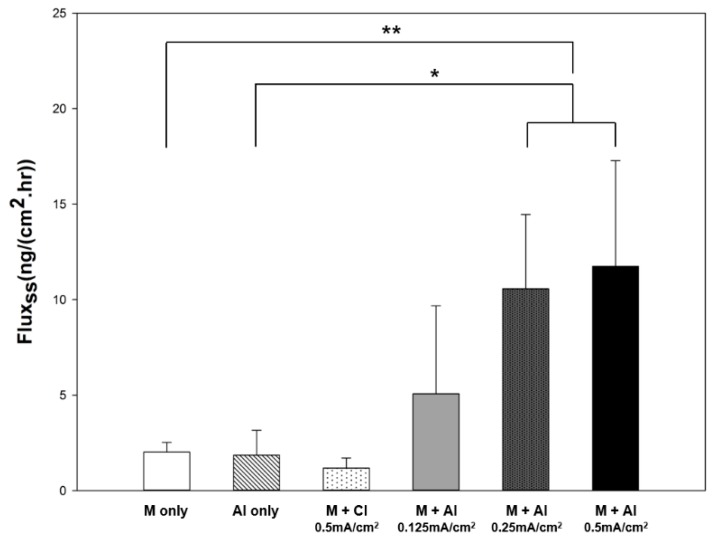
Steady-state flux of rhGH through rat skin tissue in all study groups (* or ** indicates statistically significant difference (*t*-test; *p* < 0.05 or *p* < 0.01, respectively) between treatment groups).

Microneedles painlessly pierce the main barrier of the skin, creating microchannels that increase skin permeability of small molecules and macromolecules. Iontophoresis promotes drug delivery through electrical driving forces [2]. However, only small-area microchannels are produced by microneedles, so other enhancers are needed to increase skin permeability. Because a limitation of iontophoresis is that it cannot disrupt the main barrier of the skin, a high permeation-enhancing effect is needed and achieved through combination with microneedle pre-treatment. The combination of microneedle pre-treatment and iontophoresis resulted in steady-state flux approximately 6-fold greater than that achieved in single application groups, showing synergetic effects on the permeation of rhGH into excised rat skin tissues (Figure 9).

## 5. Conclusions

In this study, we evaluated the effect of iontophoresis on the transdermal delivery of rhGH, a macromolecular model drug and the combined effect of new types of microneedles. During the iontophoresis optimization process, anodal iontophoresis with higher current density produced a greater permeation-enhancing effect. Furthermore, the combination of microneedle pre-treatment and iontophoresis showed synergetic effects. In conclusion, the combination of iontophoresis and microneedle treatment is a potential strategy for the enhancement of transdermal delivery of biopharmaceutical drugs.
